# Cortical excitability correlates with seizure control and epilepsy duration in chronic epilepsy

**DOI:** 10.1002/acn3.383

**Published:** 2017-01-19

**Authors:** Adam D. Pawley, Fahmida A. Chowdhury, Chayanin Tangwiriyasakul, Bryan Ceronie, Robert D. C. Elwes, Lina Nashef, Mark. P. Richardson

**Affiliations:** ^1^ Department of Basic and Clinical NeuroscienceKing's College LondonLondonUnited Kingdom; ^2^ Centre for EpilepsyKing's College HospitalLondonUnited Kingdom

## Abstract

**Objective:**

Cortical excitability differs between treatment responders and nonresponders in new‐onset epilepsy. Moreover, during the first 3 years of epilepsy, cortical excitability becomes more abnormal in nonresponders but normalizes in responders. Here, we study chronic active epilepsy, to examine whether cortical excitability continues to evolve over time, in association with epilepsy duration and treatment response.

**Methods:**

We studied 28 normal subjects, 28 patients with moderately controlled epilepsy (≤4 seizures per year) and 40 patients with poorly controlled epilepsy (≥20 or more seizures per year). Resting motor threshold (RMT), active motor threshold (AMT), short‐interval intracortical inhibition (SICI), intracortical facilitation (ICF) and cortical silent period (CSP) were measured, using transcranial magnetic stimulation (TMS). Disease and treatment covariates were collected (age at onset of epilepsy, epilepsy duration, number of drugs prescribed, total drug load, sodium channel drug load).

**Results:**

RMT and AMT were higher in patients than in normal subjects; RMT and AMT were higher in poorly controlled than moderately controlled patients. ICF at 12 msec and 15 msec were lower in poorly controlled patients than in normal subjects. Long‐interval intracortical inhibition (LICI) at 50 msec was higher in poorly controlled compared to moderately controlled patients. These differences were not explained by antiepileptic drug (AED) treatment or duration of epilepsy. RMT and AMT increased with duration in the poorly controlled group, but did not increase with duration in the moderately controlled group.

**Interpretation:**

Cortical excitability differs markedly between moderately controlled and poorly controlled patients with chronic epilepsy, not explained by disease or treatment variables. Moreover, the evolution of cortical excitability over time differs, becoming more abnormal in the poorly controlled group.

## Introduction

Epilepsy is a condition in which the inhibition–excitation balance in brain networks is altered in such a way that seizures can periodically emerge. Given that epilepsy may reflect an imbalance between excitation and inhibition, Transcranial Magnetic Stimulation (TMS) has been used extensively to measure cortical excitability in human epilepsy, both to investigate disease phenomena and to investigate the mechanism of action of antiepileptic drugs (AEDs).^1^ TMS has identified differences in cortical excitability between patients with epilepsy taking AEDs and healthy controls in both generalized^2^, ^3^, ^4^ and focal epilepsy^5^, ^6^; however, findings have been inconsistent. The variability of findings between studies is likely to reflect differences in AED treatment^7^, differences in seizure frequency^8^ and differences in epilepsy duration.^9^

One group of investigators used TMS to study a large set of patients with drug‐naïve new‐onset epilepsy^8^ and followed them for 3 years.^9^ The key finding of this important study was that patients with new‐onset epilepsy showed increased cortical excitability measured with TMS, which is in accord with a simple notion that epilepsy is caused by ‘hyperexcitable’ brain networks. Moreover, patients who became seizure‐free on AED treatment showed a change in TMS measurements, losing the ‘hyperexcitable’ profile and becoming normal; whereas patients who did not respond to AEDs remained ‘hyperexcitable’ for up to 3 years, despite being on AED treatment. This work suggests that patients with longstanding uncontrolled epilepsy with duration longer than 3 years may continue to show a ‘hyperexcitable’ profile, despite being on AEDs. However, this is substantially out of keeping with existing studies which show that patients with longstanding uncontrolled epilepsy have reduced cortical excitability compared to normal subjects, evidenced for example by increased motor threshold.^4^, ^6^, ^10^, ^11^

In the current study, we use TMS to investigate a group of patients with longstanding uncontrolled epilepsy. We test the hypothesis that patients with longstanding uncontrolled epilepsy have a ‘hyperexcitable’ profile of TMS measurements. We carefully examine the influence of multiple other factors, particularly seizure frequency, AED treatment load, and epilepsy duration.

## Methods

### Subjects

Patients were recruited from the epilepsy clinics at King's College Hospital, London UK, and were a consecutive series who fitted the inclusion and exclusion criteria and were able to participate. Adult patients over 18 years of age with epilepsy currently treated with AEDs were recruited into two groups: patients with between 1 and 4 seizures in the last 12 months comprised the ‘moderately controlled’ group; patients with 20 or more seizures in the previous 12 months comprised the ‘poorly‐controlled’ group. We chose these ranges of seizure frequency for the following reasons. A prior study found approximately half of patients with epilepsy had zero seizures in the previous year, approximately a quarter had 1–9 seizures in the previous year, and the rest had 10 or more^12^, regarding these latter two groups as moderately controlled active epilepsy and poorly controlled active epilepsy. We were especially concerned to minimize misclassification of subjects into moderately controlled and poorly controlled groups resulting from inaccuracy of self‐reporting of seizures^13^, therefore we purposefully created a wide separation between our groups by setting 4 as the upper limit for the moderately controlled group and 20 as the lower limit for the poorly controlled group. Patients were excluded if they had contraindications to TMS procedures, any other neurological or psychiatric condition, nonepileptic seizures, were unable to give consent, could not cooperate with TMS procedures, or did not keep a seizure diary. The study was approved by Bromley Research Ethics Committee (reference 12/LO/0230). Written informed consent was obtained in all cases.

Normal control data were available from a previously published study collected by members of the same team of investigators, using the same equipment and laboratory^14^; 28 subjects had suitable data.

### Acquisition of TMS data

Data were collected on a single occasion with subjects relaxed and alert. Electrodes were applied to the first dorsal interosseous bilaterally. The optimal coil position on the scalp for obtaining motor evoked potentials (MEPs) from the contralateral first dorsal interosseous was established.^15^ Resting motor threshold (RMT) and active motor threshold (AMT) were measured.^15^ AMT was recorded while subjects squeezed a manometer at 20% of each individual's maximum contraction force. Short‐interval intracortical inhibition (SICI) and intracortical facilitation (ICF) were measured, using conditioning‐test stimuli pairs given in a random order at each interstimulus interval (ISI; SICI at ISIs 2 msec and 3 msec, ICF at 12 msec and 15 msec). The conditioning stimulus was 80% of AMT and the suprathreshold stimulus 120% RMT. Long‐interval intracortical inhibition (LICI) was measured using two suprathreshold pulses at 120% of RMT, at ISIs of 50 msec, 150 msec, 200 msec and 250 msec. Finally, cortical silent period (CSP) was measured with single pulses applied at 120% of AMT, with subjects squeezing a manometer at 20% of their maximum voluntary contraction.

The calculation of SICI and LICI utilized custom scripts to measure the amplitudes of conditioned MEPs and to express SICI, ICF and LICI at each ISI as a percentage of the amplitude of the unconditioned MEPs (conditioned mean/unconditioned mean). To measure CSP, to minimize observer bias, we measured from the TMS stimulus artifact to the end of the CSP as indicated using the cumulative sum approach.^16^

### Potentially confounding factors and covariates

We collected the following clinical data which we assumed may associate with TMS measurements and/or associate with the assignment of the patients to moderately controlled and poorly controlled groups: age at time of TMS study, sex, age at onset of epilepsy, duration of epilepsy, epilepsy syndrome, onset lateralization in focal cases, AEDs currently prescribed, and doses.

Due to the impact of AEDs on TMS parameters^7^, ^17^, we particularly sought to take account of AED effects on TMS measurements. We adopted three measures of AED effects: number of AEDs currently prescribed; total drug load; and sodium‐channel drug load. Drug load for an individual drug was determined as the ratio of prescribed daily dose to defined daily dose. Defined daily dose is determined by the WHO Collaborating Centre for Drug Statistics Methodology^18^, defined as the assumed average maintenance dose per day for a drug used for its main indication in adults. Data for defined daily dose were accessed at http://www.whocc.no/atc_ddd_index/. Total drug load was calculated for each patient by summing the drug load for each AED. Separately, we calculated the sodium channel load for each patient. AEDs included were phenytoin, carbamazepine, lamotrigine, oxcarbazepine, zonisamide, rufinamide, lacosamide, and eslicarabazepine. Other medications such as topiramate and valproate whose probable or partial mechanism of action may include blockade of sodium channels were not included.^19^ The sodium channel drug load was calculated for each patient by summing the drug load for each sodium channel blocking AED.

### Statistical analysis

We conducted a small number of primary analyses followed by a series of exploratory secondary analyses. All statistical tests were conducted using SPSS version 21 (IBM). All TMS measures showed strong correlations between hemispheres across subjects and no significant differences between hemispheres within subjects; therefore, we averaged data from left and right for each subject. This reduced the number of nonindependent comparisons being made. For some exploratory secondary analyses, we split ipsilateral and contralateral hemispheres in the focal epilepsy group.

We assumed RMT and AMT would be highly correlated within subjects, therefore we included both measures in a single ANOVA with the within‐subjects factor “RMT‐AMT” (2 levels), and the between‐subjects factor “group” (3 levels: moderately controlled, poorly controlled, normal subjects). We tested the hypothesis that the groups differed. Any significant effects were explored using *T*‐tests to examine for differences between pairs of groups, correcting for unequal variances, and correcting for 6 comparisons (3 between group comparisons for each of 2 measures, Bonferroni‐corrected *P*‐value 0.05/6 = 0.0083). Significant ANOVA effects were also examined by constructing a series of further ANOVAs comparing moderately controlled versus poorly controlled patients, each ANOVA including one of several potentially confounding covariates (age, age of onset of epilepsy, duration of epilepsy, number of AEDs currently prescribed, total drug load, sodium channel drug load). Additionally, an ANOVA was constructed with the factor focal/generalized epilepsy.

Similarly, we assumed SICI and ICF would be correlated, therefore undertook an exactly analogous approach by including all measures in an ANOVA with the within‐subjects factor “ISI” (4 levels: 2 msec, 3 msec, 12 msec, 15 msec), and the between‐subjects factor “group” (3 levels: moderately controlled, poorly controlled, normal subjects). We tested the hypothesis that the groups differed. As with motor threshold, we further explored any significant effects using *T*‐tests, correcting for 12 comparisons (Bonferroni‐corrected *P*‐value threshold 0.05/12 = 0.0042). Significant ANOVA effects were also examined by constructing a series of further ANOVAs comparing moderately controlled versus poorly controlled patients, each ANOVA including one of several potentially confounding covariates, as for motor threshold. LICI data were examined exactly as SICI/ICF data. CSP data were examined using a univariate ANOVA but otherwise with an identical scheme (Bonferroni correcting three between‐group *T*‐test *P*‐values using a *P*‐value threshold of 0.05/3 = 0.0167).

The final primary analysis was a series of regression analyses, to examine the correlation between RMT, AMT, SICI, ICF, LICI, CSP, and the various covariates (age, age of onset of epilepsy, duration of epilepsy, number of AEDs currently prescribed, total drug load, sodium channel drug load), to test the hypothesis that TMS measures vary in association with these disease and treatment variables.

Where primary analyses had found effects of interest in the entire patient group, in subsequent secondary analyses we explored whether these effects were present in the generalized and focal groups separately. We also undertook detailed exploration of interactions between factors and covariates of greatest interest in the primary analyses.

## Results

96 subjects were included: 28 normal subjects, 28 subjects with moderately controlled epilepsy and 40 patients with poorly controlled epilepsy (see Table 1 for demographics and clinical features). There were 19 patients with IGE/GGE (8 Juvenile Myoclonic Epilepsy (JME), 5 generalized tonic clinic seizures only, 5 Juvenile Absence Epilepsy, 1 Childhood Absence Epilepsy). There were 49 patients with focal‐onset epilepsy (2 bilateral seizure onset, 26 left onset, 16 right, 5 uncertain lateralization; 9 frontal lobe onset, 36 temporal lobe, 1 parietal lobe, 3 had uncertain lobar onset). Using chi‐squared to test for a difference in proportions of moderately controlled versus poorly controlled patients with each epilepsy syndrome, there were no differences in the proportions with different lobar localizations in focal epilepsy or different IGE syndromes. The patient groups were well‐matched except for duration of epilepsy and number of AEDs currently prescribed. Twenty‐four patients were taking carbamazepine (9 moderately controlled, 15 poorly controlled), 21 were taking lamotrigine (12, 9), 15 levetiracetam (4, 11), 10 sodium valproate (5, 5), 6 topiramate (2, 4), 3 zonisamide (2, 1), 3 lacosamide (1, 2), 2 phenytoin (0, 2), 1 tiagabine (0, 1), 1 gabapentin (0, 1) and 1 rufinamide (0, 1). Using chi‐squared to test for a difference in proportions of moderately controlled versus poorly controlled patients taking each AED, there were no significant differences between these groups. Eighteen moderately controlled and 28 poorly controlled patients had epileptiform EEG abnormalities on routine EEG, and 7 in each group had an epileptogenic abnormality on MRI; using chi‐squared to test for a difference in proportions between groups, neither of these was significantly different.

**Table 1:** Demographic information and antiepileptic drug (AED) treatment for the participants

		*N*	Gender	Age (years)		Age of onset (years)		Duration of epilepsy (years)		Number of AEDs currently prescribed		Total drug load		Sodium channel drug load	
				Mean	SD	Mean	SD	Mean	SD	Mean	SD	Mean	SD	Mean	SD
Well controlled	Generalized	10	5F	34.20	11.18	14.80	6.46	20.40	13.83	1.40	0.70	1.40	1.27	0.67	0.98
	Focal	18	11F	37.39	14.28	24.18	15.30	12.94	7.33	1.33	0.59	1.43	1.08	1.20	0.79
	Combined	28	16F	36.25	13.14	20.70	13.41	15.70	10.62	1.36	0.62	1.42	1.12	1.01	0.88
Poorly controlled	Generalized	9	7F	34.78	15.05	13.33	7.57	21.44	17.88	1.78	0.67	1.62	0.89	0.69	0.63
	Focal	31	14F	42.68	14.28	18.48	14.52	23.58	12.44	1.61	0.72	1.54	0.74	0.98	0.63
	Combined	40	21F	40.90	14.64	17.33	13.37	23.10	13.62	1.65	0.70	1.56	0.77	0.91	0.63
Normal subjects		28	15F	33.46	8.25										
				*t*	*P*	*t*	*P*	*t*	*P*	*t*	*P*	*t*	*P*	*t*	*P*
Well controlled versus poorly controlled				1.137	0.176	1.013	0.315	2.492	0.015	1.815	0.074	0.566	0.574	0.469	0.641

SD, standard deviation, F, female, *t*, value of *t*‐statistic, *P*,* P*‐value.

Not all subjects underwent all TMS measures, mostly because of minor discomfort during TMS. The number of normal subjects, moderately controlled and poorly controlled patients undergoing each measure was as follows: motor thresholds *n* = 28, *n* = 28, *n* = 40 respectively; SICI/ICF *n* = 28, *n* = 24, *n* = 24; LICI *n* = 28, *n* = 24, *n* = 21; CSP *n* = 27, *n* = 28, *n* = 39.

### Motor thresholds: primary analysis

**Figure 1:**
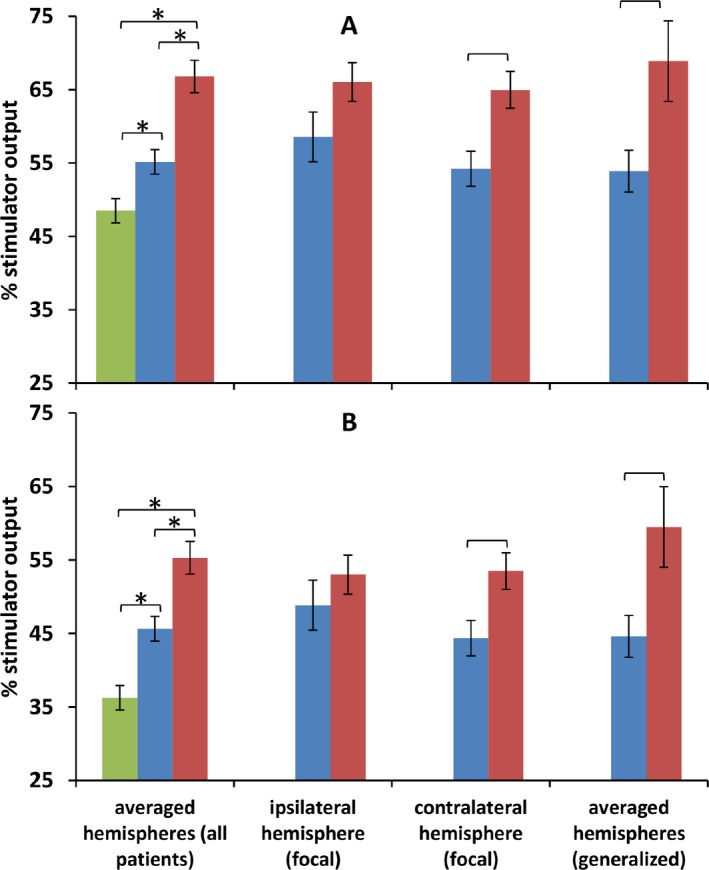
Comparison of motor thresholds between groups shows higher thresholds in poorly controlled epilepsy than moderately controlled epilepsy, indicating reduced cortical excitability in poorly controlled epilepsy. This difference was not explained by differences in treatment or disease variables. Upper panel (A) shows resting motor threshold (RMT), lower panel (B) shows active motor threshold (AMT). Normal subjects shown in green, moderately controlled subjects in blue, poorly controlled subjects in red. Comparisons indicated with a bracket and * are significant at *P* < 0.05 Bonferroni corrected, comparisons indicated with a bracket are significant at *P* < 0.05 uncorrected.

Motor thresholds differed significantly between moderately controlled patients, poorly controlled patients, and normal subjects (*F* = 26.74, *P* < 0.001, Fig. 1). Normal subjects had lower motor thresholds than either patient group (RMT vs. moderately controlled *T* = 2.819, *P* = 0.007; AMT vs. moderately controlled *T* = 4.726, *P* < 0.001; RMT vs. poorly controlled *T* = 6.528, *P* < 0.001; AMT vs. poorly controlled *T* = 7.651, *P* < 0.001). Poorly controlled patients had higher motor thresholds than moderately controlled patients (RMT *T* = 4.193, *P* < 0.001, AMT *T* = 3.884, *P* < 0.001). All these *P*‐values remained significant after Bonferroni correction. Further ANOVAs comparing moderately controlled and poorly controlled groups were carried out, including potentially confounding covariates as described in the Methods. Differences between patient groups remained extremely significant despite these additional covariates or factors (*P* = 0.001 or less in all cases), and there was no main effect or interaction involving the factor focal/generalized epilepsy. In particular, differences in motor thresholds between moderately controlled and poorly controlled groups were not explained by any AED treatment variables.

We explored the correlations between motor thresholds and several covariates as described in the Methods. Both RMT and AMT were correlated with duration of epilepsy (*r* = 0.326, *P* = 0.007; *r* = 0.309, *P* = 0.011 respectively) but no other correlations were significant (all *r* < 0.2 and *P* > 0.1). The correlation between RMT or AMT and duration of epilepsy remained significant in a series of regression analyses, each analysis including one of several additional independent variables: age, age of onset, number of drugs currently taken, total drug load, and sodium channel drug load; notably, none of the other variables had a significant effect in these regression analyses.

### Motor thresholds: secondary analysis

We examined the generalized and focal epilepsy groups separately using a threshold of *P* = 0.05 uncorrected (Fig. 1); therefore, these comparisons should be regarded as exploratory trends. In the generalized group, motor thresholds differed between moderately controlled and poorly controlled (*F* = 6.96, *P* = 0.017). Moderately controlled patients had lower motor thresholds than poorly controlled (RMT *T* = 2.427, *P* = 0.032, AMT *T* = 2.521, *P* = 0.030). In the focal group, motor thresholds differed significantly between moderately controlled and poorly controlled (*F* = 4.69, *P* = 0.036). Furthermore, motor thresholds differed between ipsilateral and contralateral hemispheres (*F* = 4.281, *P* = 0.045). In the contralateral hemisphere, motor thresholds were higher in the poorly controlled group than in the moderately controlled group (RMT: *T* = 3.100, *P* = 0.004; AMT: *T* = 2.827, *P* = 0.008) but there were no differences in the ipsilateral hemisphere.

**Figure 2:**
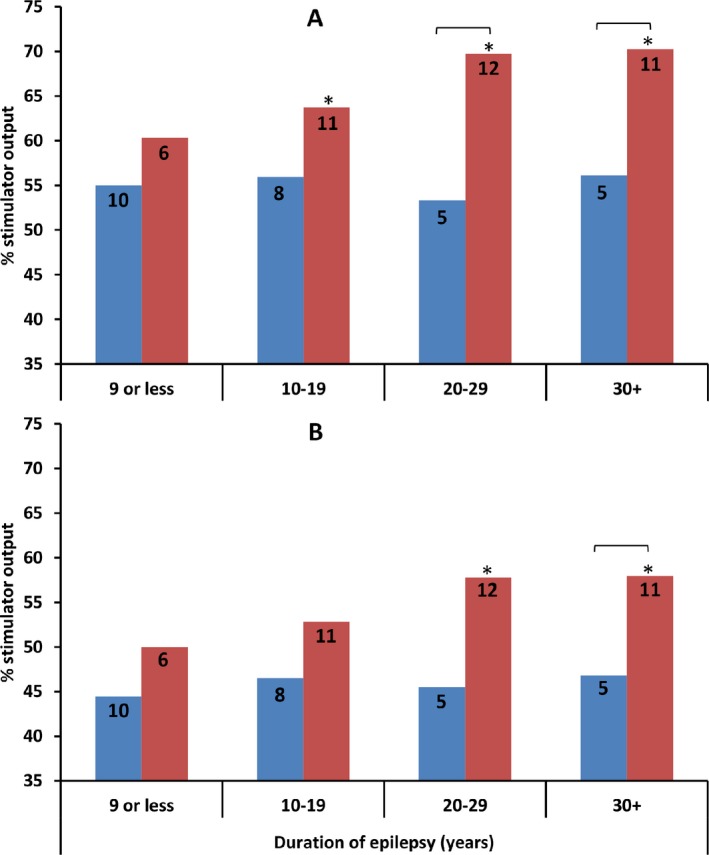
Evolution of motor threshold with duration of epilepsy differs between poorly controlled and moderately controlled epilepsy, remaining stable over time in moderately controlled but increasing over time in poorly controlled epilepsy. The correlation between motor threshold and duration was not explained by differences in treatment or disease variables. On the *x*‐axis, subjects are allocated to time bins based on epilepsy duration in years. Upper panel (A) shows resting motor threshold (RMT), lower panel (B) shows active motor threshold (AMT). Moderately controlled subjects shown in blue, poorly controlled subjects in red. Comparisons marked with a bracket show comparisons between moderately controlled and poorly controlled that are significant *P* < 0.05 uncorrected. * indicates comparisons between poorly controlled epilepsy and normal subjects that are significant *P* < 0.05 Bonferroni corrected.

Subsequently, we explored whether the correlation between motor threshold and duration of epilepsy was similar in moderately controlled and poorly controlled patients. This revealed no correlation in the moderately controlled group (correlation between duration and RMT *r* = 0.058, AMT *r* = 0.075), whereas both RMT and AMT increased with duration in the poorly controlled group (RMT *r* = 0.297, AMT *r* = 0.268). To explore these data further, we assigned each subject to one of four bins according to epilepsy duration. In each duration bin, we compared RMT and AMT between moderately controlled patients and poorly controlled patients, and compared each of these groups with normal subjects (Fig. 2); hence there were 24 nonindependent comparisons, therefore we used 0.05/24 = 0.00208 as the *P*‐value threshold. Poorly controlled patients had higher RMT in the 10–19 years, 20–29 years and 30+ years duration groups than normal subjects (*T* = 4.194, *P* = 0.001; *T* = 5.19, *P* < 0.001; *T* = 5.122, *P* < 0.001 respectively). Poorly controlled patients also had higher AMT in the 20–29 years and 30+ years duration groups than normal subjects (*T* = 5.916, *P* < 0.001; *T* = 5.519, *P* < 0.001 respectively). Moderately controlled patients did not differ from normal subjects in any duration group. Comparing moderately controlled and poorly controlled patients, at *P* = 0.05 uncorrected, poorly controlled patients had higher RMT in the 20‐29 years and 30+ years duration groups, and higher AMT in the 30+ years duration group, than moderately controlled patients (*T* = 3.702, *P* = 0.002; *T* = 2.609, *P* = 0.027; *T* = 3.07, *P* = 0.009).

### SICI and ICF: primary analysis

**Figure 3:**
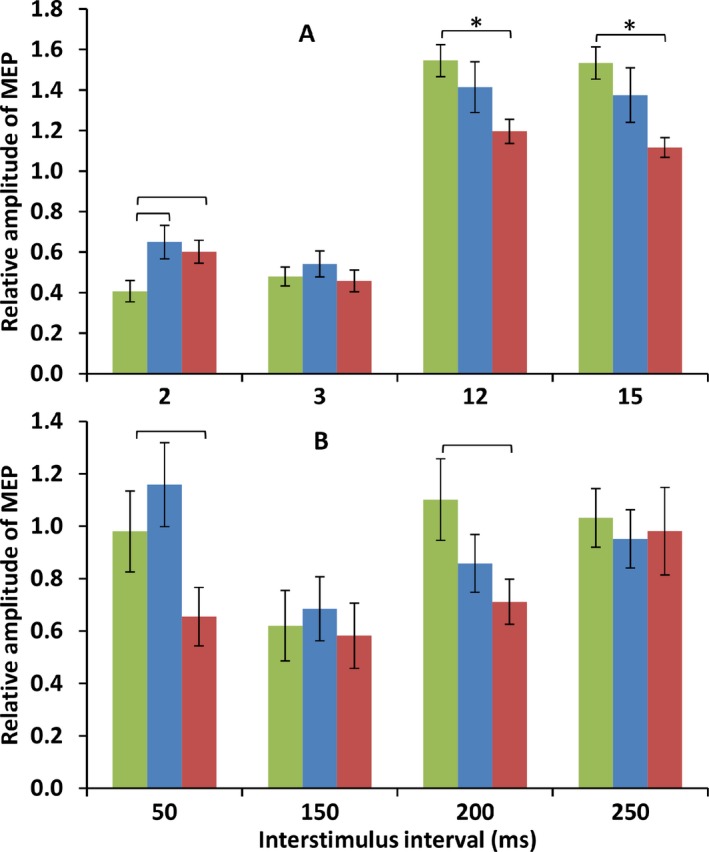
Comparison of SICI, ICF, and LICI between groups shows reduced cortical excitability in poorly controlled epilepsy. Upper panel (A) shows SICI and ICF, lower panel (B) shows LICI. On the *x*‐axis is shown the interstimulus interval in ms. Normal subjects shown in green, moderately controlled subjects in blue, poorly controlled subjects in red. Comparisons indicated with a bracket and * are significant at *P* < 0.05 Bonferroni corrected, comparisons indicated with a bracket are significant at *P* < 0.05 uncorrected. ICF, intracortical facilitation; SICI, short‐interval intracortical inhibition; LICI, long‐interval intracortical inhibition.

Comparing SICI and ICF between moderately controlled patients, poorly controlled patients, and normal subjects, although there was no main effect of group, there was a significant interaction between group and ISI (*F* = 3.186, *P* = 0.006, Fig. 3). Poorly controlled patients had less ICF than normal subjects (12 msec ISI *T* = 3.516, *P* = 0.001; 15 msec ISI *T* = 4.497, *P* < 0.001); these *P*‐values remained significant after correction for 12 nonindependent comparisons. There was a trend that normal subjects had more inhibition at 2 msec ISI than either moderately controlled or poorly controlled patients, significant at *P* < 0.05 but not surviving correction for multiple comparisons (normals vs. moderately controlled *T* = 2.480, *P* = 0.017; normals vs. poorly controlled *T* = 2.531, *P* = 0.015).

We explored the correlations between measures of SICI and ICF and several covariates as described in the Methods. ICF at 12 msec ISI and 15 msec ISI was correlated with sodium channel drug load (12 msec ISI *r* = 0.317, *P* = 0.028; 15 msec ISI *r* = 0.328, *P* = 0.023). Note that the difference in ICF between groups was not explained by sodium channel drug load. No other correlations were significant or close to significant.

### SICI and ICF: secondary analysis

We examined the generalized and focal epilepsy groups separately using a threshold of *P* = 0.05 uncorrected; therefore, these comparisons should be regarded as exploratory trends. Group sizes were smaller than for motor thresholds, especially the generalized group, and these data were generally noisier, so effects were weaker. In the generalized group, there were no differences between moderately controlled, poorly controlled and normal subjects for any ISI, although ICF at 15 msec ISI showed a weak trend to be diminished in poorly controlled patients vs. normal subjects (*T* = 2.350, *P* = 0.059). In the focal group, we split the data into ipsilateral and contralateral hemispheres. In the focal group, poorly controlled patients differed from normal subjects at 2 msec (reduced inhibition), 12 msec and 15 msec (reduced facilitation) in the ipsilateral hemisphere (*T* = 2.636, *P* = 0.013; *T* = 4.799, *P* < 0.001; *T* = 5.471, *P* < 0.001 respectively). Effects at the same ISIs were similar but weaker in the contralateral hemisphere (*T* = 2.278, *P* = 0.029; *T* = 3.246, *P* = 0.002; *T* = 3.441, *P* = 0.001 respectively). In contrast, moderately controlled patients showed fewer differences from normal subjects, with reduction in ICF at 12 msec and 15 msec in the ipsilateral hemisphere only (*T* = 2.569, *P* = 0.021; *T* = 3.980, *P* < 0.001). Moreover, there was a greater reduction in ICF at 15 msec ISI in the ipsilateral hemisphere in poorly controlled than in moderately controlled patients (*T* = 2.111, *P* = 0.047).

### LICI: primary analysis

Comparing LICI between groups, although there was no main effect of group, there was a significant interaction between group and ISI (*F* = 3.205, *P* = 0.006, Figure 3). Poorly controlled patients had inhibition at 50 ms ISI whereas moderately controlled patients did not (*T* = 2.582, *P* = 0.014); also, poorly controlled patients tended to have inhibition at 200 ms ISI whereas normal subjects did not (*T* = 2.199, *P* = 0.034); neither of these comparisons survived correction for 12 multiple comparisons. The difference between well and poorly controlled patients at 50 ms ISI was explored further, examining potentially confounding covariates as described in the Methods using a series of ANOVAs; in all instances, effects remained significant at *P* < 0.05.

We explored the correlations between measures of LICI and several covariates as described in the Methods. LICI at 200 msec ISI was correlated with sodium channel drug load (*r* = 0.311, *P* = 0.038). No other correlations were significant or close to significant.

### LICI: secondary analysis

We examined the generalized and focal epilepsy groups separately using a threshold of *P* = 0.05 uncorrected; therefore, these comparisons should be regarded as exploratory trends. Group sizes were smaller than for motor thresholds, especially the generalized group, and these data were generally noisier, so effects were weaker. In the generalized group, there were no differences between moderately controlled, poorly controlled and normal subjects for any LICI ISI. In the focal group, we split the data into ipsilateral and contralateral hemispheres. There were no differences between moderately controlled and poorly controlled groups, although LICI at 50 msec showed a trend to being increased in ipsilateral and contralateral hemispheres of poorly controlled patients (*T* = 1.749, *P* = 0.097; *T* = 2.085, *P* = 0.063, respectively). Furthermore, LICI at 200 msec ISI was increased in moderately controlled and poorly controlled patients compared to normals (*T* = 2.168, *P* = 0.040; *T* = 2.114, *P* = 0.041).

### CSP

CSP differed significantly between groups (*F* = 10.375, *P* < 0.001). Both patient groups had significantly longer CSP than normal subjects (moderately controlled vs. normal *T* = 2.933, *P* = 0.005; poorly controlled vs. normal *T* = 4.006, *P* < 0.001) but there was no difference between patient groups (*T* = 0.146, *P* = 0.885). Therefore, although this measure differed between treated epilepsy and normal subjects, it did not reveal any differences between moderately controlled and poorly controlled epilepsy, and was therefore not explored further.

## Discussion

In this study, we found that motor threshold was higher in patients with poorly controlled epilepsy than moderately controlled epilepsy, counterintuitively suggesting that cortical excitability is lower in poorly controlled epilepsy than moderately controlled. This difference in motor threshold could not be explained by any differences between groups in age, age of onset of epilepsy, epilepsy duration, or epilepsy type (focal or generalized). Crucially, we found that this difference between moderately controlled and poorly controlled epilepsy was not explained by differences in AED treatment. We found that motor threshold was higher in patients with poorly controlled epilepsy than moderately controlled epilepsy in generalized and focal epilepsy, although in the focal group this effect was seen most strongly in the contralateral hemisphere. Of particular note, we found that motor threshold increased with duration of epilepsy, which could not be explained by age, age of onset of epilepsy, or AED treatment. This relationship between epilepsy duration and motor threshold appeared to be confined to the poorly controlled group, in which motor threshold increased with duration. In contrast, motor threshold did not increase with duration of epilepsy in the moderately controlled group. At the shortest duration of epilepsy (≤9 years duration), motor threshold in the poorly controlled group did not differ significantly from motor threshold in the moderately controlled group or the normal control group.

We found that ICF was diminished in poorly controlled epilepsy but not in moderately controlled, again counter‐intuitively suggesting that cortical excitability is lower in poorly controlled epilepsy than moderately controlled. Furthermore, we found that the reduction of ICF in poorly controlled patients, compared to moderately controlled, was more easily detected in focal than generalized patients, although this reduction was not absent in the generalized patients. Moreover, the reduction in ICF was more marked in the ipsilateral hemisphere in focal patients. In addition, we found that LICI at 50 msec and 200 msec was enhanced in poorly controlled epilepsy but not in moderately controlled, once again counterintuitively suggesting that cortical excitability is lower in poorly controlled epilepsy than moderately controlled. This difference between groups was not explained by any differences in epilepsy duration, epilepsy type (focal or generalized), or drug treatment. The increase in LICI at 50 msec in poorly controlled patients was more easily detected in the focal patients, although was not absent in the generalized patients.

Our finding of increased motor threshold in AED‐treated epilepsy patients is similar to previous findings in both focal^6^, ^10^ and generalized epilepsy^4^, ^11^. However, other studies have found reduced thresholds in patients, but these studies were typically in drug naïve new‐onset patients^3^, ^20^, ^21^. We propose that reduced cortical excitability in longstanding poorly controlled epilepsy is a pathophysiological feature of epilepsy, and not due to AED treatment. Moreover, we propose that the increase in motor threshold over time in poorly controlled epilepsy is related to an as yet unknown pathophysiological factor that evolves slowly over years, and is not due to AED treatment.

It might appear difficult to reconcile our findings with previous studies which found increased cortical excitability in new‐onset patients whose seizures did not come under control with AEDs, compared to new‐onset patients who became seizure‐free on AEDs^8^, ^22^. In particular, we found that poorly controlled patients with long‐standing epilepsy had higher motor thresholds than moderately controlled, whereas these previous studies found that poorly controlled patients with new‐onset epilepsy or epilepsy for up to 3 years had lower motor thresholds than seizure‐free.^8^, ^9^ Crucially, our data strongly suggest that motor thresholds steadily increase over time in the poorly controlled group, whereas thresholds remain stable over time in the moderately controlled. Therefore, our data would not rule out the possibility that motor thresholds could be lower in poorly controlled than seizure‐free epilepsy in the early disease course.^8^, ^22^ Our data suggest that over time there is a marked drop in cortical excitability in poorly controlled epilepsy, such that poorly controlled patients have higher thresholds than moderately controlled patients later in the disease course.

In this study, we did not attempt to examine the relationship between TMS measures and specific seizure types. Although potentially of interest, such a study would be challenging for several reasons, all of which are the consequence of relying entirely on the patient's ability to provide a detailed history. Firstly, it is often difficult, on the basis of the patient's history, to distinguish between focal seizures that involve or do not involve a disturbance of consciousness (i.e. between seizures previously termed simple and complex partial). Secondly, it is sometimes difficult, on the basis of the patient's history, to distinguish between a severe focal seizure with many motor features and collapse versus a bilateral convulsive seizure. Thirdly, and crucially, in order to determine whether the occurrence of specific seizure types determines changes in TMS measures over a very long period of time, we would need a completely accurate record of all seizure types occurring over a period of up to several decades, and for this record to be sufficiently detailed to include seizures that occurred rarely (e.g., a bilateral convulsive seizure occurring decades ago in a patient with temporal lobe epilepsy) or seizures that are often missed (eg. absences in patients with juvenile myoclonic epilepsy); such an accurate and complete record is very rarely available.

At this point, we cannot provide a detailed understanding of exactly which mechanistic features of the epileptic brain TMS is able to detect. Although simplistic descriptions of TMS measurements often use terms such as ‘inhibition’ and ‘excitation’ in a manner suggesting that underlying mechanisms of TMS effects are understood, the detailed underlying mechanisms are not known. There are some informative models explaining how TMS stimuli may be converted to a motor output and how that output may be modulated by drugs and by paired‐pulse stimulation protocols.^23^, ^24^ The models have in common that TMS motor output is determined by the membrane potentials of neurons having input to cortical layer 5 pyramidal neurons, the nature of the input (inhibitory or excitatory), the number of inputs (synapses), and the membrane potential of the pyramidal neurons. Although more detailed understanding is missing at the current time, it is plausible that this set of neurons and connections is highly relevant to the epileptic process. Although none of our patients had seizure onset in a focal region of the motor cortex, finding physiological abnormalities in a brain region remote from putative seizure onset zones is increasingly accepted as part of the network hypothesis of epilepsy.^25^, ^26^

Although it is a widely used method, using peripheral EMG as an index of cortical excitability measured with TMS is somewhat indirect; increasingly, EEG is being collected concurrently with TMS in order to assess cortical excitability from a more direct readout.^27^, ^28^, ^29^ In a study of focal‐onset patients, it was shown that abnormal EEG phenomena could be induced by focal TMS stimulation during periods of otherwise normal EEG, whereas no such abnormal phenomena could be induced in healthy normal subjects, suggesting that enhanced excitability of the epileptic brain can be revealed by TMS‐EEG.^27^ A subsequent study in IGE revealed similarly that epileptiform discharges could be induced by TMS, and also that TMS could unmask covert states of hyperexcitability in which discharges were more likely to occur.^28^ Furthermore, TMS‐EEG may allow these abnormal responses to be anatomically mapped into the underlying epileptic brain network.^29^ A future study using TMS‐EEG to examine the effects of epilepsy severity and duration may therefore cast light on the underlying pathophysiological mechanisms.

An important question, not addressed by this study, is whether the increase in motor threshold over time in the poorly controlled group is due to progressive brain atrophy affecting the poorly controlled more than the moderately controlled group. It is well established that an increased distance between skull and motor cortex is associated with an elevated motor threshold, with each millimetre of increased distance increasing the RMT by 2–4%.^30^, ^31^, ^32^ To account for the observations made here, the distance between skull and motor cortex in the poorly controlled group would need to be approximately 4–8 mm greater than in the well‐controlled group. We do not have imaging data to address this question, but this additional degree of atrophy seems substantial. Moreover, although some studies show an association between atrophy and number of seizures^33^, ^34^, other studies have shown similar atrophy in well‐controlled patients^35^, ^36^. A cross‐sectional study suggests that atrophy progresses more quickly in patients with a duration of epilepsy >14 years.^37^ Nevertheless, to conclusively determine whether the differences in motor threshold observed in this study are due to differences in cortical excitability specifically, these measures could be obtained in patients with neuroimaging so that any group differences in scalp‐cortex distance and/or cortical thickness can be accounted for.

It may seem surprising that similar effects could be observed across a very wide range of epilepsy syndromes and AED treatments. However, at least three arguments support the proposition that there are unifying mechanistic features across the range of epilepsies that could allow similar effects across epilepsy syndromes and AEDs. Firstly, although numerous genetic and acquired abnormalities are associated with seizures and epilepsy, nonetheless, there may be unifying mechanisms at the macroscale that are common to different microscale causes. Theoretical modelling work based on seizures from several epilepsy models suggests that, across the range of epilepsy types, a small set of system parameters are responsible for seizure onset, evolution, offset and recurrence.^38^ Secondly, evidence suggests that both focal and generalized seizures have their onset in localized microcircuits,^39^ which may be epileptogenic because of specific connectivity patterns or motifs that are similar across different types of epilepsy.^40^ Thirdly, the existence of broad‐spectrum AEDs suggests that similar mechanisms prevent seizure onset across a wide range of epilepsy syndromes.^41^ Therefore, we argue that there are multiple underlying microscale mechanisms of epilepsy, but at the macroscale there is a limited set of rules governing the dynamics of seizure onset and offset, and a limited set of mechanisms to stabilize seizure networks.

We believe the study described here, and previous studies^8^, ^9^, have identified a feature of the epileptic brain, similar across syndromes, that evolves very slowly over a timescale of years, and differs fundamentally between well‐controlled and poorly controlled epilepsy.

## Author Contributions

A.D.P. contributed to conception and design of the study, acquisition and analysis of data, drafting a significant portion of the manuscript or figures. F.A.C. also contributed to conception and design of the study, acquisition and analysis of data. C.T. contributed to acquisition and analysis of data. B.C. also helped in the acquisition and analysis of data.L.F: conception and design of the study, acquisition of data. M.P.R contributed to conception and design of the study, acquisition and analysis of data, drafting a significant portion of the manuscript or figures.

## Conflict of Interest

The authors report no conflicts of interest.

## Supporting information


**Figure S1:** SICI and ICF at ISIs of 2, 3, 12 and 15 msec.
**Figure S2:** LICI at ISIs of 50, 100, 200 and 250 msec.
