# Supplementary material for: Cortical excitability correlates with seizure control and epilepsy duration in chronic epilepsy
Source: Ann Clin Transl Neurol. 2017 Jan 19;4(2):87–97. doi: 10.1002/acn3.383 (PMC5288462; doi:10.1002/acn3.383)
Supplement: Supplementary file 1 — Figure S1: SICI and ICF at ISIs of 2, 3, 12 and 15 msec. Figure S2: LICI at ISIs of 50, 100, 200 and 250 msec. [file ACN3-4-87-s001.docx]

**Supplementary Figures**


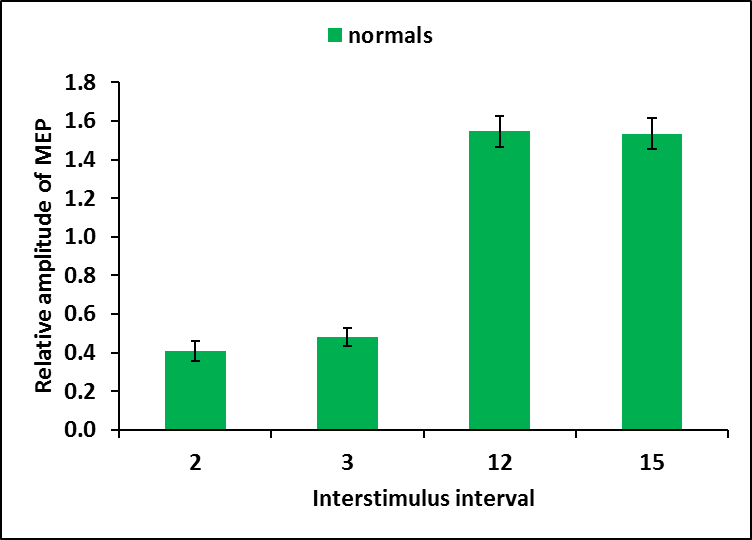

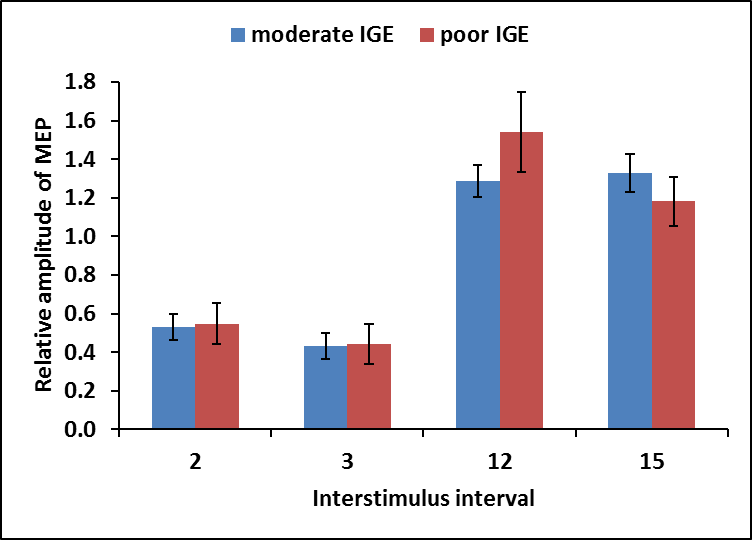

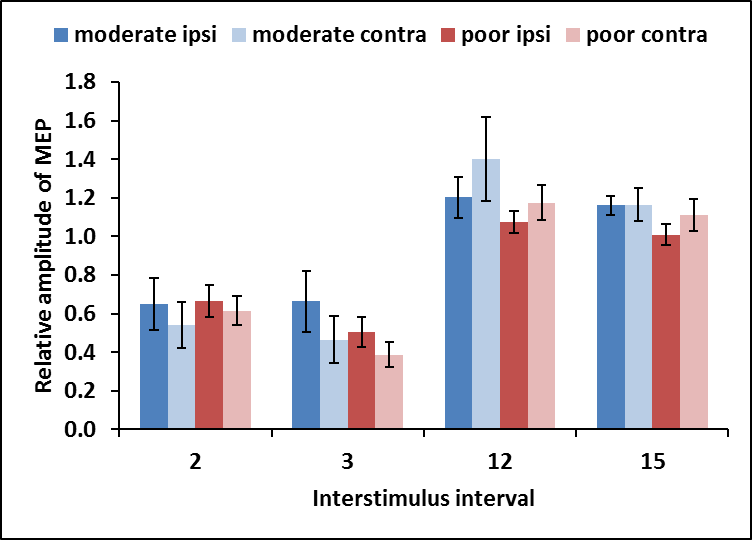


**Supplementary Figure 1** (above): SICI and ICF at ISIs of 2, 3, 12 and 15ms. Left panel – normal subjects (green bars). Middle panel – patients with IGE, divided into moderately controlled (blue bars) and poorly controlled (red bars). Right panel – patients with focal epilepsy, divided into moderately controlled ipsilateral hemisphere (solid blue bars), poorly controlled ipsilateral hemisphere (solid red bars), moderately controlled ipsilateral hemisphere (faint blue bars), and poorly controlled ipsilateral hemisphere (faint red bars). Error bars are +/- 1 SEM.


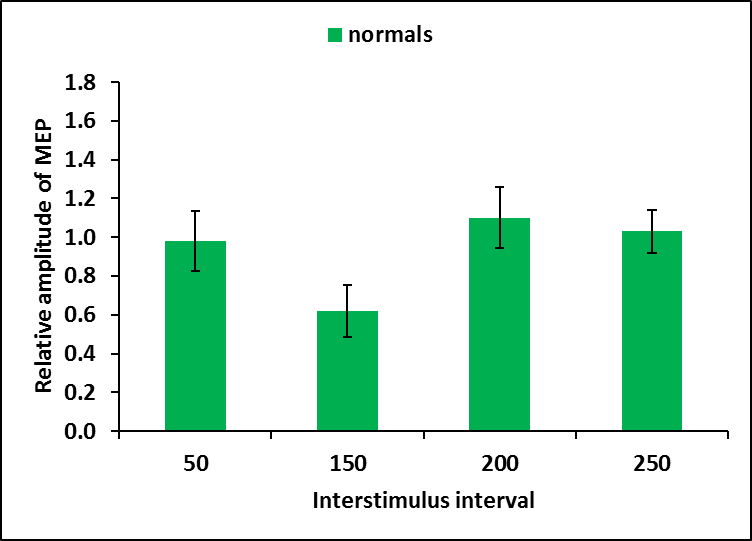

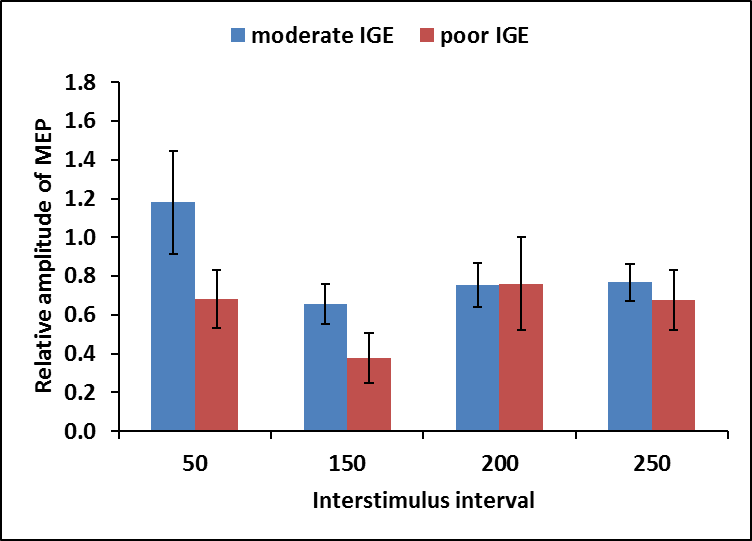

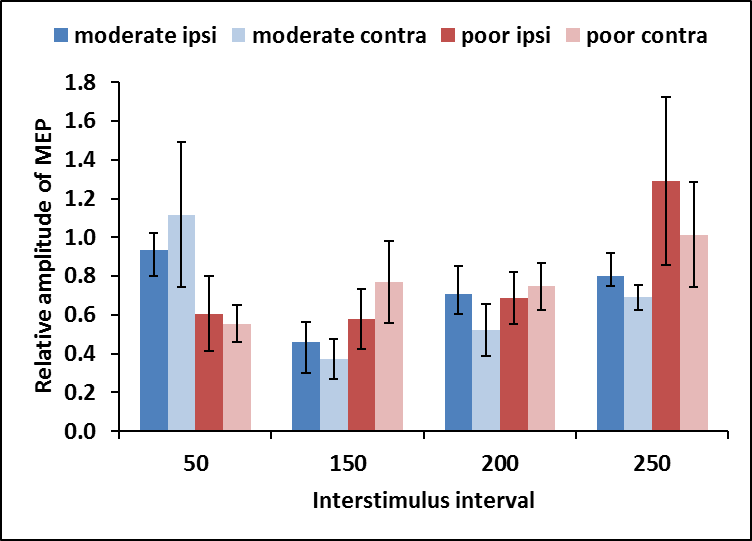


**Supplementary Figure 2** (above): LICI at ISIs of 50, 100, 200 and 250ms. Left panel – normal subjects (green bars). Middle panel – patients with IGE, divided into moderately controlled (blue bars) and poorly controlled (red bars). Right panel – patients with focal epilepsy, divided into moderately controlled ipsilateral hemisphere (solid blue bars), poorly controlled ipsilateral hemisphere (solid red bars), moderately controlled ipsilateral hemisphere (faint blue bars), and poorly controlled ipsilateral hemisphere (faint red bars). Error bars are +/- 1 SEM.
